# Bioaccessibility and antioxidant activity of β-carotene loaded nanostructured lipid carrier (NLC) from binary mixtures of palm stearin and palm olein

**DOI:** 10.1016/j.heliyon.2022.e08913

**Published:** 2022-02-09

**Authors:** Miftakhur Rohmah, Anton Rahmadi, Sri Raharjo

**Affiliations:** a Department of Agricultural Technology, Faculty of Agriculture, Mulawarman University, Jl. Paser Balengkong Kampus Gunung Kelua, Samarinda, 75119, Indonesia; b Research Center for Drugs and Cosmetics from Tropical Rain Forest (PUI-PT Oktal), University of Mulawarman, Samarinda 75123, Indonesia; c Department of Food and Agricultural Product Technology, Faculty of Agricultural Technology, Universitas Gadjah Mada, Bulaksumur, Yogyakarta 55281, Indonesia

**Keywords:** β-carotene, Nanostructured lipid carriers (NLC), Palm stearin, Palm olein, Bioaccessibility, Antioxidant activity

## Abstract

β-carotene (βC) is an essential nutrient for health. It is a potent antioxidant, anti-cancer, and anti-inflammatory substance. However, βC has high hydrophobicity property, indicating a low absorption level in the digestive tract. The bioavailability of βC is reasonably low. Lipid-based delivery systems such as nanostructured lipid carriers (NLC) potentially can help to overcome this problem. This research evaluated the bioaccessibility of the nanostructured mixture of palm stearin (PS) and palm olein (PO) and the antioxidant activity of βC in the structure. β-carotene bioaccessibility was studied by measuring the micellization during *in vitro* digestion. Antioxidants activity was measured by 2.2′-azino-bis (3-ethylbenzothiazoline- 6-sulphonic acid) (ABTS) and 2, 2 – diphenyl -1- picrylhydrazyl (DPPH) reduction methods. *In vitro* gastrointestinal digestion model indicated that nanostructured lipid carrier enhanced bioaccessibility and antioxidants activity of βC. This suggests that the formulated NLC system can be used effectively to deliver lipophilic bioactive such as βC in beverage products.

## 1 Introduction

Lipid-based systems are one of the common methods for carrying non-polar bioactive with the aim of increasing the solubility of these components. Lipid-based delivery system is capable of increasing bioaccessibility and antioxidant activity of lipophilic bioactive substances by using various types of lipids such as Medium Chain Triglycerides (MCT), oleic acid, glyceryl behenate, glyceryl palmitostearate, glyceryl monostearate/monostearate, cetyl palmitate, stearic acid, wax and other types of lipids (Tamjidi et al., 2013).

β-carotene is an antioxidant and colorant used in food items (Mezzomo and Ferreira, 2016). β-carotene is an antioxidant (Sy et al., 2012), anticancer (Gloria et al., 2014), and anti-inflammatory (Kawata et al., 2018). β-carotene is an essential vitamin for preserving eye health and lowering cardiovascular disease risk (Maria et al., 2015). Its breakdown, isomerization, oxidation, and gastrointestinal tract alteration (Boon et al., 2010) make it difficult to use as a functional food ingredient. Due to its low water solubility (C Log P = 17.62), β-carotene absorption is inefficient and very variable (Gul et al., 2015). However, a lipid-based delivery method can circumvent these β-carotene constraints (Pan et al., 2016).

The delivery system is expected to increase the solubility and stability of the bioactive substances. Consequently, these bioactive substances can be more easily absorbed in the digestive system, as indicated by their increased bioaccessibility. Recent research has effectively encapsulated another potent antioxidant (α-tocopherol) in polymer-lipid hybrid nanoparticles with rapid release for 2 h and controlled release for 24 h (Rizwanullah et al., 2021). Incorporation of βC in the form of NLC is a way to improve its absorption in the digestive system. The simulated gastrointestinal tract model is widely used as a simplified method to determine the release performance of NLC delivery system (Gonçalves et al., 2021; Dima et al., 2020; Park et al., 2018; Qian et al., 2012). Several limitations of simulated digestive system are acknowledged in comparison to the *in vivo* system, i.e., the simulated system is prepared in static conditions and may not reflect the dynamic conditions *in vivo*. However, static condition in a controlled environment is preferable to provide valuable preliminary and complementary data to *in vitro* models (Goncalves et al., 2021; Martínez-Ballesta et al., 2018).

Nanotechnology research is currently on-demand, a nano delivery system such as nanostructured lipid carriers (NLC). The NLC is a potential lipid-based carrier system for encapsulation, protection, and carrying water-insoluble bioactive components (de Souza Simões et al., 2017; Patra et al., 2018). The NLC is developing and improving a Solid Lipid Nanoparticle (SLN) delivery system produced from solid-lipids doped in water. SLN has disadvantages because of the low diffusion rate, which requires a long release time (Tapeinos et al., 2017). The NLC, as the second generation of a lipid delivery system, has a composition consisting of a mixed matrix of solid-lipids, liquid-lipids in water stabilized with surfactants (Nobari Azar et al., 2020). The NLC has high encapsulation and controlled release abilities. It is thermodynamically stable, while bioactive components bioavailability is observed (Tamjidi et al., 2013; Rohmah et al., 2020a; Rohmah et al., 2020b; Elmowafy and Al-Sanea, 2021).

However, the reports on the application of lipids derived from palm oil fractionation of palm stearin (PS) as solid lipid and palm olein (PO) as liquid lipid is still very limited. In the light of the previous studies (Rohmah, 2020a, 2020b), PS and PO with Tween 80 surfactant can be used to deliver micronutrients via the NLC system. While the fatty acids compositions of PS and PO were similar, their respective proportion influenced their physicochemical properties and crystallinity. In addition, the NLC formulated using combination of PS and PO also improved micronutrient delivery system (Rohmah et al., 2020a). The optimal βC-NLC formulation shows an inverse relationship between particle size and efficiency encapsulation of βC. The Differential Scanning Calorimetry-Thermal Gravimetry analysis demonstrates that βC-NLC has a high thermal stability. According to the Franz diffusion model, the optimized βC-NLC exhibits favorable diffusion properties (Rohmah, 2020b). However, the biological activity of this delivery system is yet to be reported. This article aimed to observe bio-accessibility of βC loaded into NLC using a simulated *in vitro* digestion system and to evaluate its antioxidant activity using DPPH and ABTS methods.

## 2 Materials and methods

### 2.1 Materials

β-carotene (97.0% purity; Sigma-Aldrich, St. Louis, MO). The excipients used in the NLC preparation were the following: palm stearin and palm olein from PT. Smart. Tbk (Surabaya, Indonesia), Tween 80 (Merck, Darmstadt, Germany), α-amylase (taka-diastase from aspergillus oryzae, Product # 86250, _1.5 units/mg), mucin (from the porcine stomach), bovine serum albumin (98%), pepsin (Pepsin from porcine gastric mucosa, Product #P7000, 800–2,500 units/mg protein), pancreatin (pancreatin porcine pancreas, Product #P8096, 1 _ USP specifications), lipase (lipase type II, crude from porcine pancreas, Product #L3126), and bile salt extract (B8631, porcine) were also purchased from Sigma-Aldrich (St Louis, MO, USA). The BHT was used as standard (Sigma-Aldrich, UK), α-tocopherol (ATP) standard (Sigma-Aldrich, UK), ABTS (2.2′-azino-bis (3-ethylbenzothiazoline-6-sulphonic acid) (Sigma-Aldrich, UK), βC standard (Sigma-Aldrich, UK), DPPH (2, 2 – diphenyl -1- picrylhydrazyl) (Sigma-Aldrich, UK). All other chemicals used were of analytical grade.

### 2.2 Preparation of β-carotene loaded nanostructured lipid carriers

The βC-NLC were prepared by the high shear hot homogenization method (Zhu et al., 2015; Naseri et al., 2015). The lipid phase consisted of PS and PO (5.5:4.5 w/w ratio). The βC concentration was 250 μg/mL dissolved at 60 °C in PS and PO mixture (Rohmah et al., 2020b). An aqueous surfactant of Tween 80 (1:4.9 w/w ratio to lipid phase) was heated up to the same temperature as the molten lipid phase. The hot surfactant solution was poured onto the hot lipid phase, and homogenization was carried out at 24000 rpm (30 min) using an Ultra-Turrax (IKA T25 digital Ultra–Turrax Germany). The final βC-NLC consisted of 24% lipid-surfactant and 76% PBS water (w/w). The NLC was sonicated using a Branson 1800M (Dietzenbach, Germany) at 20 kHz for 15 min and then left to cool to room temperature (27 ± 2 °C). Composition and Process of making βC NLC, βC-emulsion, βC -Tween, βC-PBS are explained in Table 1. Blank NLC were prepared using similar method, except without the βC.

**Table 1:** Composition and Process of making βC NLC, βC-emulsion, βC -Tween, βC-PBS.

Components	βC NLC	βC emulsion	βC -Tween	βC -PBS
PS-PO (lipid phase ratio)	5.5:45 w/w	5.5:45 w/w	-	-
BC concentration	250 μg/mL	250 μg/mL	250 μg/mL	250 μg/mL
Tween 80	1:4.9 w/w (lipid: surfactant ratio)	1:4.9 w/w (lipid: surfactant ratio)	0.01%	-
Lipid + surfactant	26% of final solution (w/w)	26% of final solution (w/w)	-	-
PBS Water (pH = 7)	76% PBS water (w/w)	76% PBS water (w/w)	Added until 100 mL of mixture	Added until 100 mL of mixture
Process	Homogenized with ultra-turrax at 24,00 rpm, 30 min Sonicated at 20 kHz, 15 min	Homogenized with magnetic stirrer at 600 rpm, 30 min		

### 2.3 Simulated gastrointestinal tract model

*In vitro* simulated digestion was performed to determine bioaccessibility (Qian et al., 2012). An *in vitro* gastrointestinal tract model (GIT), consisted of mouth, gastric and intestinal phases, was used to simulate biological fate of ingested food samples. The composition of the salivary fluid, duodenal and intestinal gastric are listed in Table 2. Subsequently the *in vitro* digestion simulation was conducted as follow.

a. Mouth Phase:

**Table 2:** Constituents and concentrations of the various simulated juices used in the *in vitro* digestion model.∗

Components	Saliva	Gastric juice	Duodenal juice	Bile juice
Inorganic components	10 ml KCl 89.6 g/l 10 ml KSCN 20 g/l 10 ml NaH2PO4 88.8 g/l 10 ml Na2SO4 57 g/l 1.7 ml NaCl 175.3 g/l 20 ml NaHCO3 84.7 g/l	15.7 ml NaCl 175.3 g/l 3.0 ml NaH2Po4 88.8 g/l 9.2 ml KCl 89.6 g/l 18 ml CaCl2.2 H2O 22.2 g/l 10 ml NH4Cl 30.6 g/l 6.3 ml HCl 37 % g/g	40 ml NaCl 175.3 g/l 40 ml NaHCO3 84.7 g/l 10 ml KH2PO4 8 g/l 6.3 ml Kci 89.6 g/l 10 ml MgCl2 5 g/l 180 μl HCl 37% g/g	30 ml NaCl 175.3 g/l 68.3 ml NaHCO3 84.7 g/l 4.2 ml KCl 89.6 g/l 150 μl HCL 37 % g/g
Organic components	8 ml urea 25 g/l	10 ml glucose 65 g/l 10 ml glucuronic acid 2 g/l 3.4 ml urea 25 g/l 10 ml glucosamine hydrochloride 33 g/l	4 ml urea 25 g/l	10 ml urea 25 g/l
Add to the mixture of organic + inorganic components	290 mg α-amylase 2.5 g pepsin 25 mg mucin	1 g BSA 15 mg uric acid 3 g mucin	9ml CaCl2.2H2O 22,2 g/l 1 g BSA 9 g pancreatin 1.5 g lipase	10 ml CaCL2.2H2O 22.2 g/l 1.8 g BSA 30 Embed

∗ Qian et al. (2012).

A sample of 5 mL was put in a closed bottle, mixed with α-amylase, and vortexed. The electrolyte solution was added as much as 6 mL. The solution was then incubated in a water bath shaker for 5 min at a speed of 100 rpm and a temperature of 37 °C.

b. Gastric phase:

After 5 min, the mouth phase was continued in the gastric phase by mixing pepsin and vortexed. An electrolyte stock solution of 12 mL was added and incubated for 2 h in a water bath shaker at a speed of 100 rpm and a temperature of 37 °C. Hence, the sampling time point for the gastric phase was 120 min after the initial time.

c. Intestinal phase:

After incubation for 2 h in the gastric phase, the intestinal phase was followed by mixing pancreatin, lipase, and bile enzymes. The mixtures were then vortexed. Intestinal electrolyte stock solution was added as much as 18 mL and incubated for 2 h in a water bath shaker at a speed of 100 rpm and a temperature of 37 °C. Hence, the sampling time point for the intestinal phase was 240 min after the initial time.

### 2.4 *In vitro* release and bioaccessibility β-carotene

Measurement of βC in the sample initially, after exposure to the mouth, stomach, and intestinal phases, was quantified by the UV-visible spectroscopy method (Qian et al., 2012). The βC was extracted using ethanol (1: 2 v/v), vortexed, and centrifuged at 14000 rpm, temperature 25 °C for 60 min. The ethanol phase containing βC was separated and measured its absorbance at ƛ 454 nm, using a UV-vis spectrophotometer. Pure ethanol was used as a blank. A standard curve of βC was prepared to quantify the βC levels in the sample at a concentration of 0–20 ppm.

The βC-NLC bioaccessibility was measured using a simulated *in vitro* digestion carried out on samples and expressed as percent micellization, i.e., the proportion of carotenoids incorporated in micelles compared to the content of initial carotenoids digested. After digestion, samples were separated from the micellar phase following the method of (Salvia-Trujillo et al., 2013) with modifications. After the sample passed the GIT simulation, then the digesta from the small intestine phase was centrifuged at 15000 rpm, at 25 °C for 60 min. The middle part, which is the aqueous phase, is assumed to be the micellar phase. The micellar phase is filtered using a syringe filter, the filtrate was then used to determine the concentration of βC by the UV-visible spectroscopy method (Qian et al., 2012). According to the equation: Bioaccessibility = 100 x (C_filtered_/C_rawdigesta_), is determined where C_filtered_ and C_rawdigesta_ are the concentrations in the micelle fraction and the total collected after the small intestine phase, respectively.

### 2.5 Antioxidant activity using DPPH radical scavenging assay

Sample (0.1 mL) were mixed with 3.9 mL of 0.025 % DPPH solution, and the mixture was shaken vigorously, followed by incubation for 30 min at 28 °C. The absorbance was measured at 517 nm by utilizing UV spectrophotometry. Methanol was used as a blank. The BHT, α-tocopherol, dan βC were used as the standard. The percentage of antioxidant activity was calculated based on the difference between the blank and the sample absorbances divided by the blank absorbance. Antioxidant activity, which was expressed as 50 % reduction of free radical DPPH^•^ (IC_50_), was determined using linear regression (Kedare and Singh, 2011).

### 2.6 Antioxidant activity with ABTS radical scavenging assay

ABTS^+•^ radical cation was generated by reacting 2 mM of ABTS^+•^ and 2.45 mM of potassium persulfate in water and incubating the resulting mixture for 12–16 h at room temperature in the dark conditions. Ethanol was used as a blank. The BHT, α-tocopherol, and βC were used as standards. The measurement of free radical binding activity was carried out by reacting 3 mL of test reagent with 1 ml of the sample, then incubated at 30 °C for 30 min. The absorption of ABTS blank solution was carried out at a wavelength of 734 nm by UV spectrophotometer. The percentage of antioxidant activity was determined based on calculating the difference between the blank and the sample absorbances divided by the blank absorbance. Antioxidant activity, which was expressed as 50 % reduction of free radical DPPH• (IC_50_), was determined using linear regression (Shirwaikar et al., 2011).

### 2.7 Data analysis

All measurements were made on at least two freshly prepared samples, and each sample was measured in triplicate. The results were reported as averages and standard deviations. The differences among treatments were calculated based on an analysis of variance (ANOVA) and a post-hoc Tukey test with a confidence level of 95%. A statistical analysis software (GraphPad Prism) was used for these data analyses.

## 3 Results and discussion

**Figure 1:**
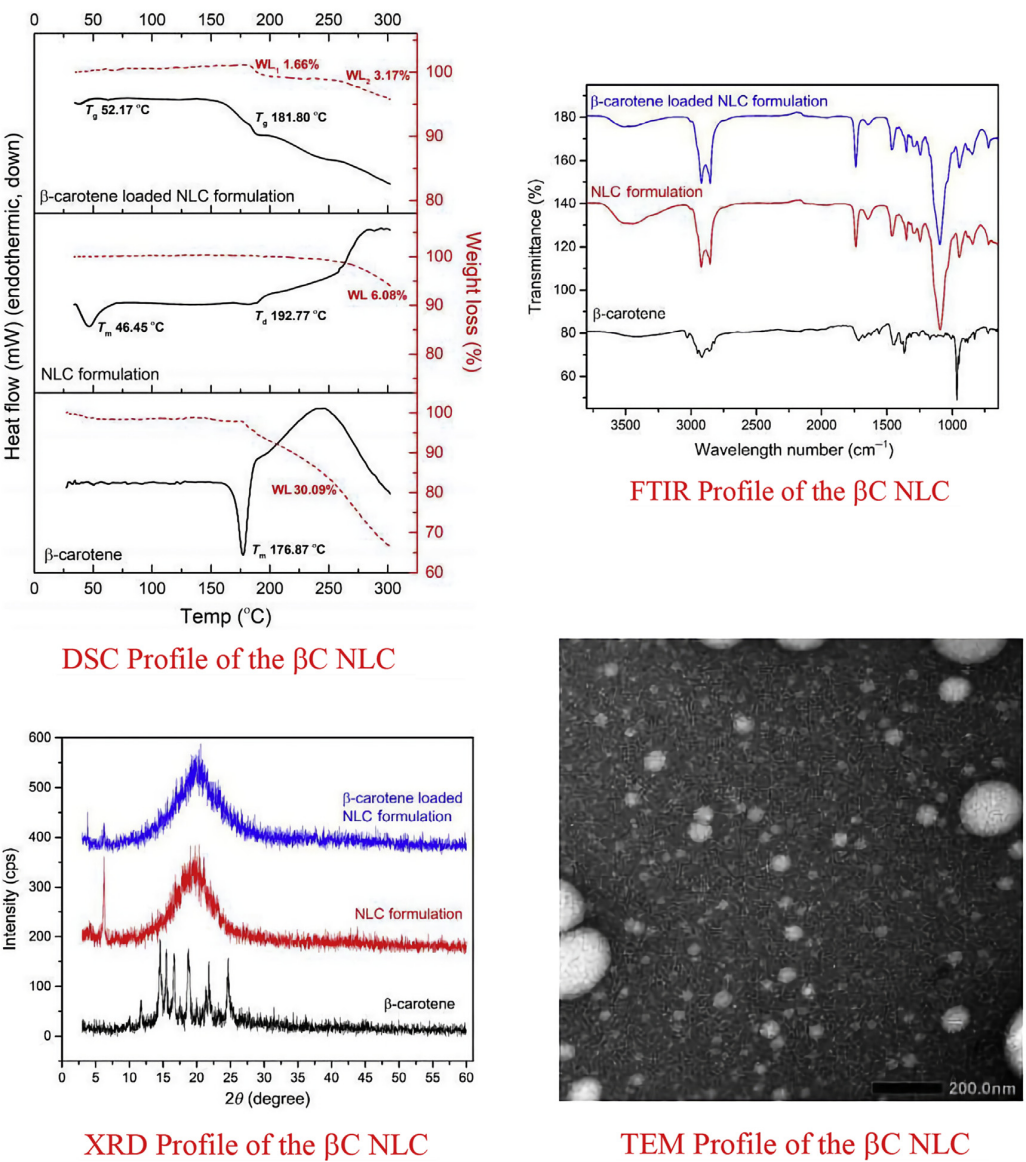
DSC, FTIR, XRD, and TEM profile of the βC NLC∗. ∗Rohmah (2020b).

β-carotene NLC for this paper was originated from an optimized formula obtained from past research Various characteristics, i.e. particle size, polydispersity index, zeta potential, encapsulation efficiency, DSC, XRD, FTIR, and TEM image were published to indicate the physicochemical profile of the resulting βC-NLC (Table 3 and Figure 1) (Rohmah et al., 2020b).

**Table 3:** Predicted optimum ranges of the independent variables and comparison of the observed and predicted values in the NLC (actual values are expressed as mean, SD, n = 3)∗.

Response variables	Predicted value	Observed value	Residual
Particle size (nm)	142.70	166.3 ± 0.19	16.54
Polydispersity index	00.26	0.35 ± 0.1	34.61
Zeta potential (mV)	−24.9	−26.9 ± 0.17	8.03
Encapsulation Efficiency (%)	91.15	91.2 ± 0.15	−0.05

The predicted levels of formulation factors obtained by the software were 5.5:4.5 (w/w) of palm stearin ratio, 1:4.9 (w/w) lipid:surfactant ratio, and 24:76% (w/w) (lipid + surfactant: water) ratio. Residual was calculated as (predicted value – observed value)/predicted value x 100%.

∗ Rohmah (2020b).

### 3.1 *In vitro* release of βC from the βC-NLC

Delivery system based on PO and PS is an effort to utilize local raw materials to increase bioaccessibility of the bioactive substance such as βC in the digestive system. In previous studies, this delivery system has been tested for its physical stability and chemical properties (Rohmah et al., 2020a, 2020b). However, how much of the active component of βC is available in a delivery system needs to be known in a simulated in vitro digestive model. This is important to do because the bioactive substances in the delivery system can be completely exposed to enzymatic reactions, mechanical digestion in the mouth, low pH in the stomach, and alkaline pH in the small intestine simulated under controlled conditions *in vitro*.

**Figure 2:**
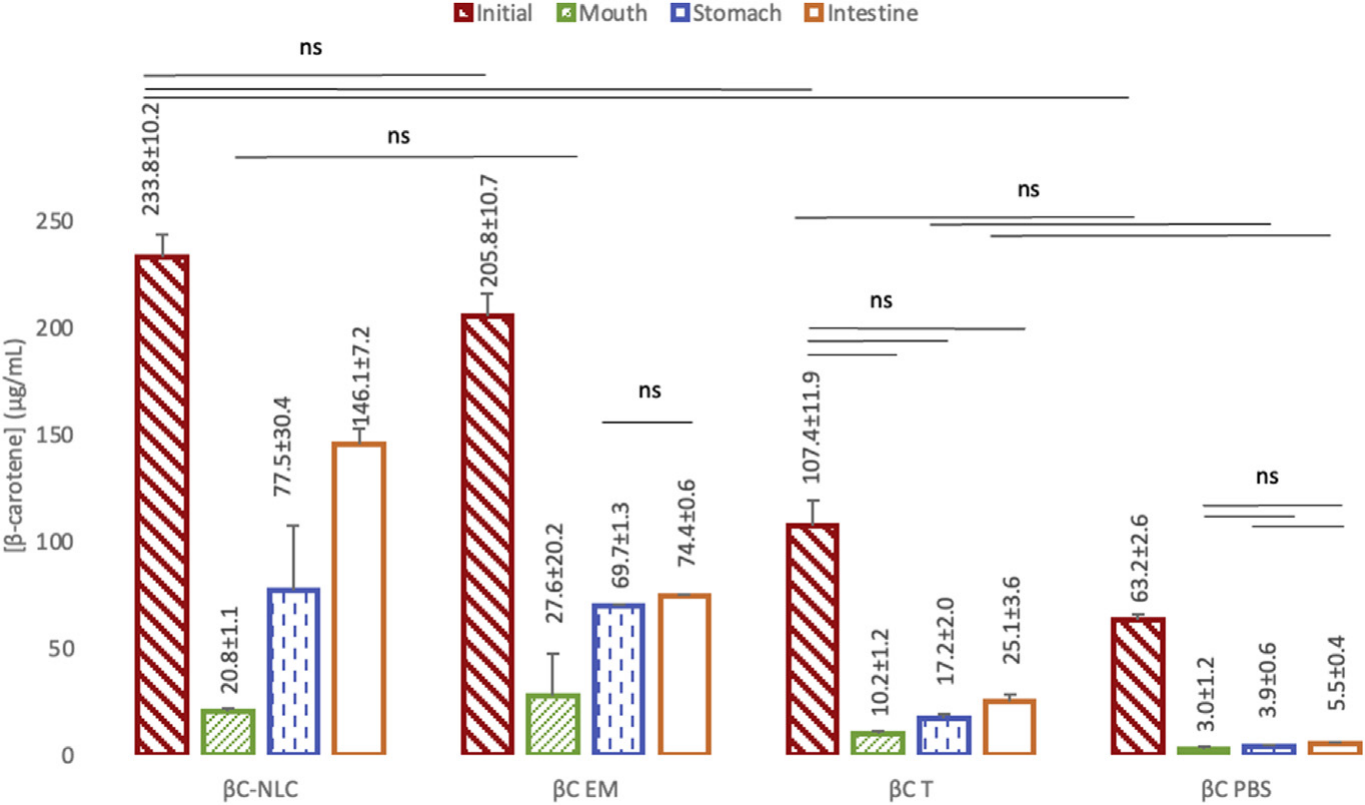
Release of βC from nanostructured lipid carrier compared to other delivery system during simulated *in vitro* digestion. βC-NLC: β-carotene loaded nanostructured lipid carriers; βC-EM: β-carotene Emulsion; βC-T: β-carotene in Tween 80 (0.01%); βC-PBS: β-carotene (Phosphate Buffered Saline pH 7 solution). All treatments are significantly different (P < 0.05) except indicated by lines with “ns”.

The initial concentrations of βC in the systems were different regardless the amount of βC added were the same (250 μg/mL) due to the solubility of βC in the delivery system. NLC can carry the most optimum βC in comparison to emulsion, tween, and PBS system. βC recovery after 240 min for each system were different due to the solubility and stability of BC in the system. However, 240 min sampling time in the simulated digestive system for βC was already optimum according to previous literatures (Hur et al., 2011). Figure 2 indicates that the bioactive compound incorporated in a delivery system is released in simulated conditions of the mouth, stomach, and the small intestine. The NLC system has a higher loading capacity for βC than that of the emulsion system because the solubility of βC in the NLC system is greater. The sustained release profile of βC was observed for βC-NLC compared to βC-Emulsion (EM), βC-Tween (T), and βC- Phosphate Buffered Saline pH 7 solutions (PBS) in the simulated *in vitro* digestion fluids in the mouth, stomach, and small intestine phases.

**Figure 3:**
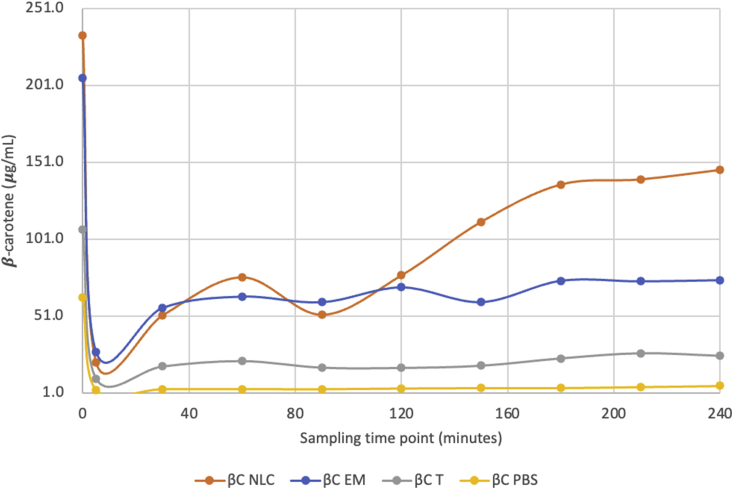
Time based β-carotene release in simulated digestive system from βC NLC, βC-emulsion, βC -Tween, and βC-PBS. βC-NLC: β-carotene loaded nanostructured lipid carriers; βC EM: β-carotene Emulsion; βC T: β-carotene in Tween 80 (0,01%); βC-PBS: β-carotene (Phosphate Buffered Saline pH 7 solution). Sampling time point for initial phase (0 min), mouth phase (5 min), stomach phase (120 min), and intestinal phase (240 min).

After 4 h of incubation, βC-NLC released in the small intestine phase (233 μg/mL) was higher than the other samples (Figure 3). The mouth phase shows a low βC release in all samples and increase during the gastric and intestinal phases. The digestion component affects the ability of βC release from the system. NLC effectively dissolves and releases βC in the small intestine, showing the system's ability to reduce the occurrence of degradation in the digestive tract. Yao et al. (2014) reported that a lipid-based delivery system success was due to the increased solubility of lipophilic bioactive compounds. Other studies have reported the success of NLC in dissolving lipophilic components such as curcumin (Park et al., 2018), essential oil (Bashiri et al., 2020), vitamin D (Rabelo et al., 2018), lycopene (Sharma et al., 2021), β-carotene (Pezeshki et al., 2019), quercetin and piperine (Chaudhari et al., 2021).

### 3.2 Bioaccessibility

**Figure 4:**
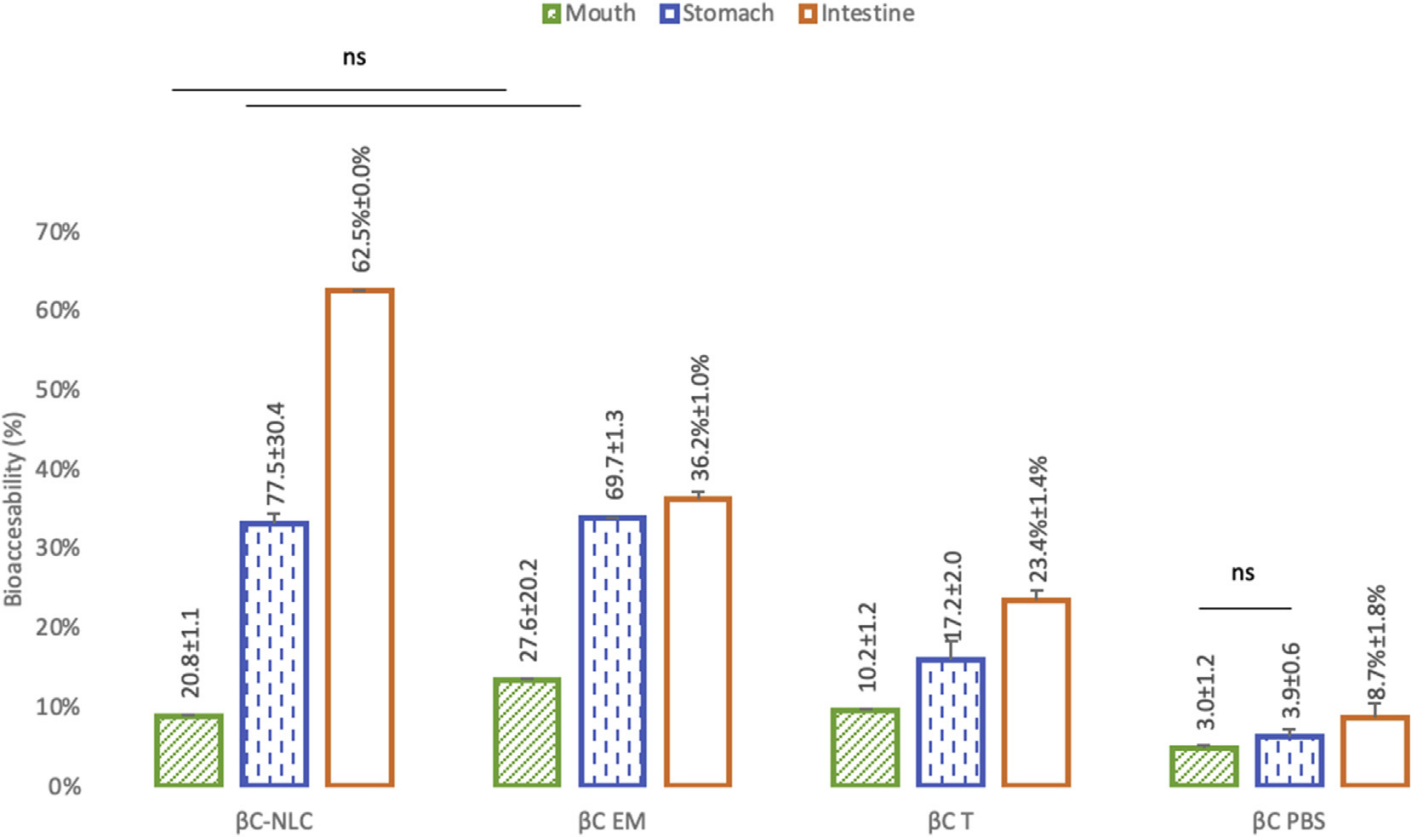
Bioaccessibility (%) of βC in different delivery system as determined after simulated *in vitro* digestion. βC-NLC: β-carotene loaded nanostructured lipid carriers; βC EM: β-carotene Emulsion; βC T: β-carotene in Tween 80 (0,01%); βC-PBS: β-carotene (Phosphate Buffered Saline pH 7 solution). All treatments are significantly different (P < 0.05) except indicated by lines with “ns”.

Direct addition of βC in the food products is very limited because of its high hydrophobicity, low bioavailability, high reactivity, and instability to heat, light, and oxygen (Brito-Oliveira et al., 2017). The *in vitro* accessibility of encapsulated βC was evaluated to determine the effectiveness of the NLC delivery of βC. Figure 4 shows that the bioaccessibility of βC-NLC was higher than that of EM, T, and PBS (60.7%, 34.1%, 23.4%, and 8.7%, respectively). For the phenomenon of bioaccessibility of βC, the effect of the βC delivery system was slightly more dominant than the effect of the digestion process (42.96% vs. 34.02%) and the interaction between the digestive system and the digestion process. For the digestive stage, all treatments were significant, except for control ßC-PBS only in the mouth and stomach.

The bioaccessibility of βC-NLC is higher due to long-chain fatty acids in PS and PO. According to (Qian et al., 2012), long-chain fatty acids are easier to form micelles with high solubility, thereby increasing bioaccessibility. This result is similar to previous research. Linoleic and oleic long-chain fatty acids derived from flaxseed can increase the bioaccessibility of lipophilic bioactive components (Sotomayor-Gerding et al., 2016). The PS and PO were dominated by palmitic acid (47–74%) and oleic acid about 40%, thus increasing the bioaccessibility of βC-NLC (Farouk et al., 2021; Pantzaris and Sue, 2017).

### 3.3 Antioxidant activity of β-carotene

**Figure 5:**
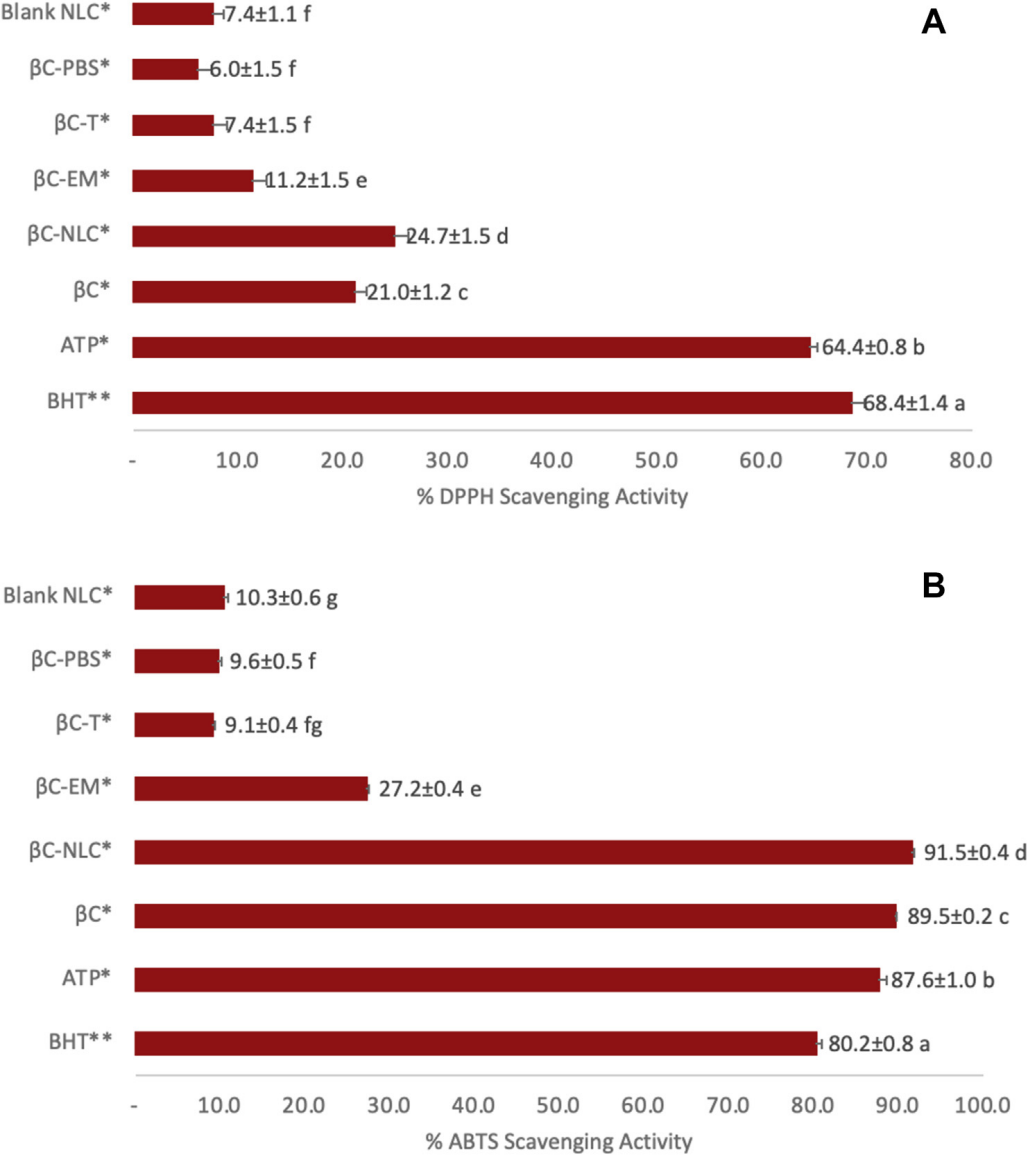
Antioxidant activity of βC-NLC compared to other βC in other delivery system as measured by (A) DPPH and (B) ABTS assay. BHT: Butylated Hydroxy Toluene; ATP: α-tocopherol; βC: β-carotene; βC-NLC: β-carotene loaded nanostructured lipid carriers; βC T; β-carotene in Tween 80 (0,01%); βCPBS; β-carotene in Phosphate Buffered Saline pH 7; BNLC: Blank nanostructured lipid carriers; at a concentration of ∗ 30 μg/mL ∗∗ 10 μg/mL.

The ßC is a powerful antioxidant, is well known as singlet oxygen quencher and scavenger peroxyl radicals, however prone to light and heat exposure (Farouk et al., 2021). Antioxidant activity was evaluated using ABTS and DPPH assays. The two methods were suitable to measure antioxidant activity in oil-based products (Christodouleas et al., 2014). The βC-NLC was comparable to PBS, T, Blank NLC, BHT, α-tocopherol, and ßC. The ßC-NLC has 91.47 ± 1.9 and 24.72 ± 0.38% free radical scavenging activity as measured by the ABTS and DPPH assay, respectively (Figure 5). There were significant differences in the percentage of βC-NLC antioxidant activity with BHT, α-tocopherol, ßC, T, PBS, and Blank NLC. However, the βC-NLC possessed a moderate to strong antioxidant activity. The ABTS assay shows relatively higher free radical scavenging activity than that of the DPPH assay. It suggests that the ABTS assay is more responsive so that it correlates positively with free radical scavenging activity compared to the DPPH assay.

Similar results reported by Prasad et al. (2011), using DPPH and ABTS assay in determining carotenoids' antioxidant activity from *Canarium odontophyllum*. It showed that the ABTS was more highly correlated than the DPPH, with free radical scavenging activity reaching 85% and 29%, respectively. Zhao et al. (2016) reported extracts of *Lycium barbarum* L. samples containing carotenoid showed a low correlation to DPPH free radical scavenging activity. This was caused by the presence of β-ionon rings, which could reduce pi-electrons' resonance effect because of a steric hindrance to lower the free radical-scavenging activity. Oil-based systems can function as secondary antioxidants, reducing the rate of lipid oxidation (Christodouleas et al., 2014).

**Figure 6:**
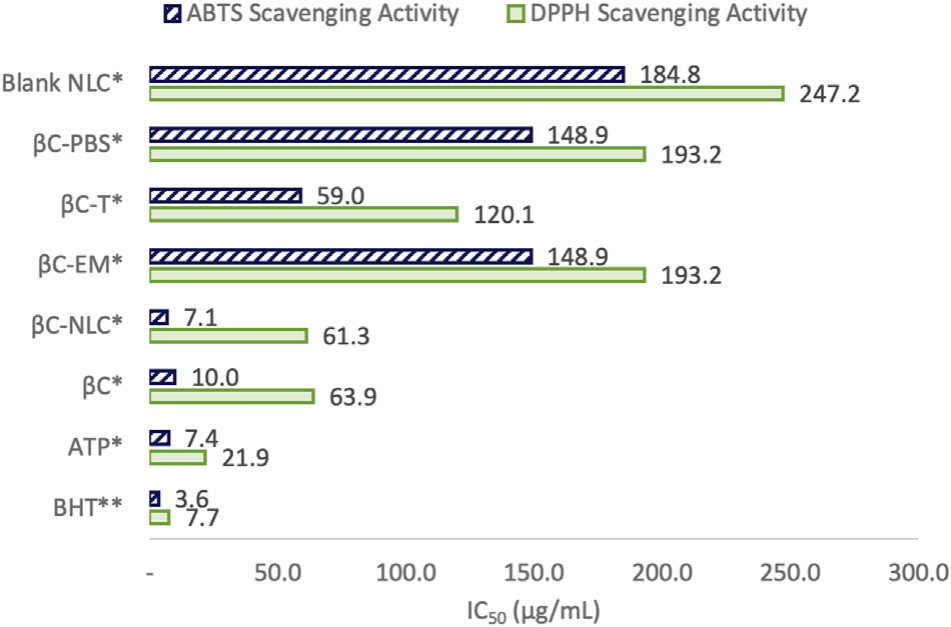
The IC_50_ of βC-NLC compared to βC in other delivery system as determined by the DPPH and ABTS assays. BHT: Butylated Hydroxy Toluene; ATP: α-tocopherol; βC: β-carotene; βC-NLC: β-carotene loaded nanostructured lipid carriers; βC T; β-carotene in Tween 80 (0.01%); βC PBS; β-carotene (Phosphate Buffered Saline pH 7 solution); Blank NLC: Blank nanostructured lipid carriers.

Figure 6 shows the IC_50_ values indicate the samples concentrations that can inhibit free radicals by 50%. A low IC_50_ value indicates greater sample ability free radical scavenging activity. The IC_50_ of βC-NLC as measured using the ABTS assay was 7.0 μg/mL. It was almost similar to ATP with an IC_50_ of 7.4 μg/mL. However, the IC_50_ of βC-NLC as measured using DPPH assay showed higher value (61.2 μg/mL) than that of α-tocopherol (21.9 μg/mL), but it was almost the same as IC_50_ of pure βC (63.9 μg/mL). Overall, the IC_50_ of BHT as measured using ABTS was lower (3.2 μg/mL) than that of the DPPH assay (7.7 μg/mL). The IC_50_ value of the NLC was lower as compared to other systems that carry βC, i.e., EM, T, and PBS. It was concluded that the βC-NLC increased antioxidant activity as compared to βC without the presence of delivery system. Similar results were reported by Rodriguez-Ruiz et al. (2018) that astaxanthin which was loaded in the NLC delivery system increased antioxidant activity as measured by the ABTS and TEAC methods. The oil-based delivery system can be secondary antioxidants with the mechanism of reducing the rate of oxidation and free radical scavenger (Christodouleas et al., 2014).

**Figure 7:**
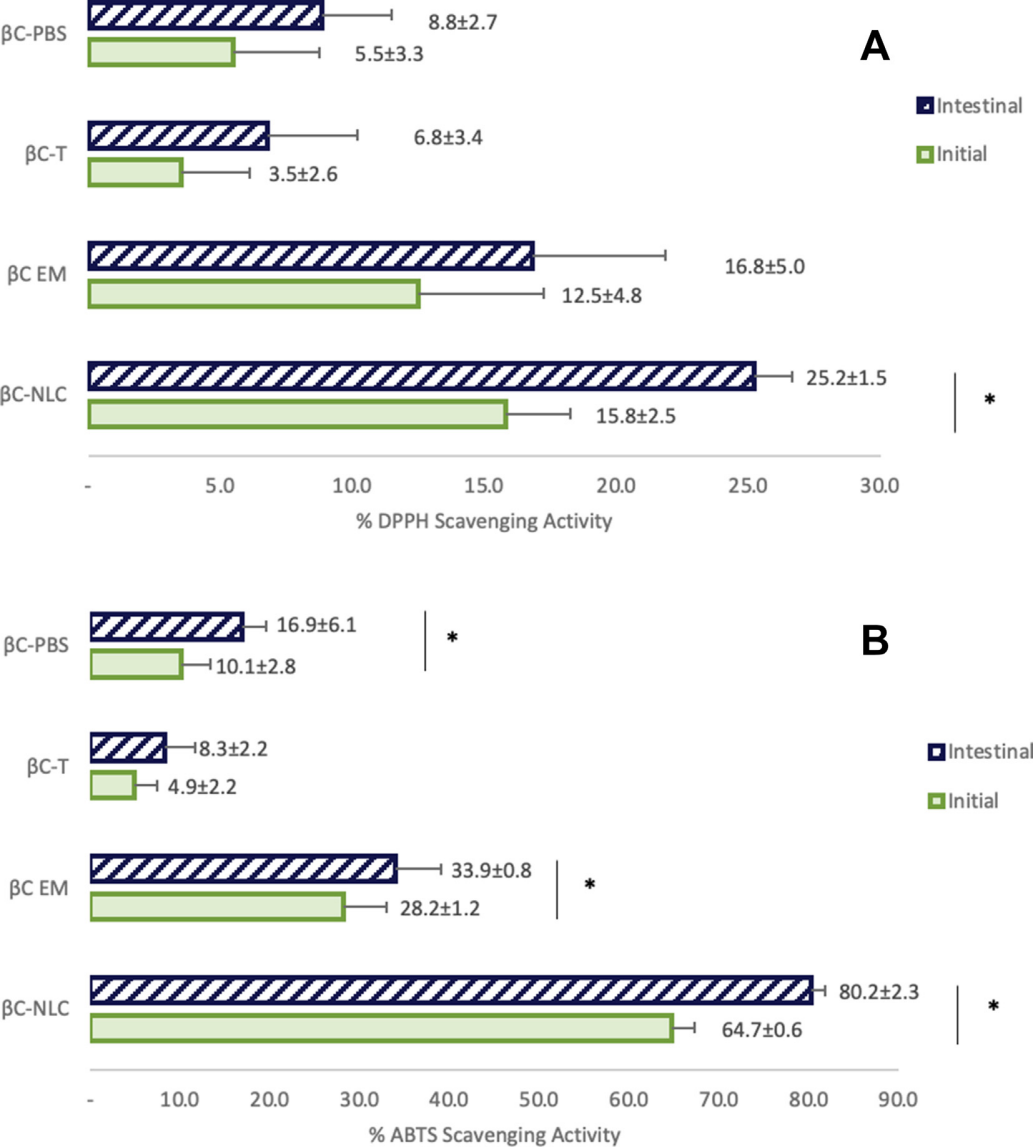
Changes in (A) DPPH and (B) ABTS free radical scavenging assay at initial and intestinal phase in simulated *in vitro* gastrointestinal digestion. βC-NLC: β-carotene loaded nanostructured lipid carriers; βC EM: β-carotene in Emulsion; βC T; β-carotene in Tween 80 (0.01%); βC PBS: β-carotene in (Phosphate Buffered Saline pH 7 solution) measurement at concentration 25 μg/mL.

The antioxidant activity assay in the simulated *in vitro* digestion system aimed to evaluate the potential antioxidant activity of the NLC delivery system at the initial and the end of the digestive process (after the mouth, stomach, and small intestine phase). The antioxidant activity of βC-NLC was compared to EM, T, and PBS samples at a concentration of 25 μg/mL. The results showed changes in DPPH and ABTS free radical scavenging activity of samples during digestive simulation (Figure 7). Antioxidant activity as measured by both the ABTS and DPPH methods showed the same trend, i.e., βC-NLC > EM > T > PBS. The ABTS radical scavenging activity of the βC-NLC at the initial phase was 64.7 ± 0.5% and increased to 80.2 ± 2.3% at the end phase in the small intestine, while the DPPH radical scavenging activity of the βC-NLC at the initial phase was 15.7 ± 2.5% and increased to 25.1 ± 1.4% at the end phase. Overall free radical scavenging activity of βC-NLC is higher than that of the controls. It may be influenced by the greater solubility of βC in the NLC delivery system and due to the nano size of NLC particle. A similar result reported by Karimi et al. (2018) that antioxidant activity of turmeric extract loaded into NLC was higher than that in the form of turmeric extract only.

## 4 Conclusion

This study showed that βC can be loaded effectively within NLC from a mixture of palm stearin and palm olein. The NLC delivery system was able to increase the bioaccessibility and antioxidant capacity of βC. The NLC system was the most suitable candidate for βC loading capacity and yielded consistent results when it was released into the simulated *in vitro* digestive system. Consequently, the bioaccessibility provided by the NLC system was superior to that of the ordinary emulsion system, especially in the simulated digestive condition of the small intestine. Antioxidant activity of βC-NLC as indicated by its IC_50_ value was equivalent to that of βC, but it was superior to free radical scavenging activity of βC in the ordinary emulsion. Overall, the increased release and antioxidant activity of βC are due to the lipid-based delivery system, such as NLC, which facilitates higher βC solubility. This study provides information on the effectiveness of the βC delivery system for food and beverage applications.

## Declarations

### Author contribution statement

Miftakhur Rohmah: Performed the experiments; Analyzed and interpreted the data; Wrote the paper.

Anton Rahmadi: Contributed reagents, materials, analysis tools or data; Wrote the paper.

Sri Raharjo: Conceived and designed the experiments; Contributed reagents, materials, analysis tools or data; Wrote the paper.

### Funding statement

This work was supported by Postdoctoral Grant of 10.13039/501100012521Universitas Gadjah Mada, Indonesia (Contract Number:6144/UN1.P.III/DIT-LIT/PT/2021).

### Data availability statement

Data included in article/supplementary material/referenced in article.

### Declaration of interests statement

The authors declare no conflict of interest.

### Additional information

No additional information is available for this paper.
